# Sodium–glucose cotransporter 2 inhibitor-induced euglycemic diabetic ketoacidosis in a patient with coronavirus disease 2019: a case report

**DOI:** 10.1186/s13256-021-03232-3

**Published:** 2022-01-04

**Authors:** Edwin Sze Sian Yii, Athirah Wan Azli, Premela Naidu Sitaram

**Affiliations:** 1 Department of Anaesthesiology and Intensive Care, Faculty of Medicine, University of Malaya, Kuala Lumpur, Malaysia; 2 Department of Anaesthesiology and Intensive Care, Hospital Sungai Buloh, Selangor Darul Ehsan, Malaysia

**Keywords:** Euglycemic diabetic ketoacidosis, SGLT-2 inhibitors, Empagliflozin, COVID-19, Diabetes mellitus

## Abstract

**Background:**

Sodium–glucose cotransporter 2 inhibitors are among the new-generation oral antihyperglycemic agents that have been used in the treatment of type 2 diabetes mellitus. With the recent coronavirus disease 2019 pandemic and rise of cases in the third wave, diagnosis of life-threatening euglycemic diabetic ketoacidosis may easily be overlooked or missed.

**Case presentation:**

We present the case of a 37-year-old Malay gentleman with underlying type 2 diabetes mellitus on empagliflozin, who presented to our hospital with symptomatic coronavirus disease 2019 infection and diabetic ketoacidosis. He developed severe rebound euglycemic diabetic ketoacidosis due to the continuous usage of empagliflozin for glycemic control alongside intravenous insulin.

**Conclusions:**

Physicians should have a high index of suspicion in diagnosing and managing euglycemic diabetic ketoacidosis, including withholding treatment of sodium–glucose cotransporter 2 inhibitors during the acute management of diabetic ketoacidosis.

## Background

Euglycemic diabetic ketoacidosis (euDKA) was first described as a discrete entity by Munro *et al*. in 1973. The report described 37 cases with a glucose concentration at presentation of less than 16.7 mmol/L (300 mg/dL) in individuals with type 1 diabetes mellitus (T1DM) [1, 2]. According to the Joint British Diabetes Societies Inpatient Care Group: The Management of Diabetic Ketoacidosis in Adults, criteria for diagnosis of diabetic ketoacidosis (DKA) require ketonemia ≥ 3 mmol/L or significant ketonuria (more than 2+ on standard urine sticks), capillary blood glucose > 11 mmol/L, serum bicarbonate (HCO_3−_) < 15 mmol/L, and/or venous pH < 7.3 [3].

Empagliflozin is a sodium–glucose cotransporter (SGLT-2) inhibitor that was approved in August 2014 by the Food and Drug Administration (FDA) to be used as an oral antihyperglycemic agent for the treatment of type 2 diabetes mellitus (T2DM) [4], and has since gained popularity due to its cardioprotective properties [5]. According to the European Medicines Agency (EMA) as of May 2015 in EudraVigilance, a total of 101 cases of euDKA had been reported worldwide in T2DM patients treated with SGLT-2 inhibitors [4]. With the recent worldwide pandemic of coronavirus disease 2019 (COVID-19), we report a case of severe euDKA in a patient with symptomatic severe acute respiratory syndrome coronavirus 2 (SARS-CoV-2) infection.

## Case presentation

A 37-year-old Malay gentleman with underlying T2DM for the past 2 years, presented to hospital at day 4 of illness with lethargic and shortness of breath, and tested positive for COVID-19 infection upon admission. There were no other gastrointestinal symptoms at initial presentation to the hospital, and he was able to take orally as usual. His initial serum glucose level and arterial blood gas (ABG) values were within normal range. He required admission for observation in view of symptomatic COVID-19 infection and was not started on any steroid therapy. He claimed to be compliant to his medications, which included empagliflozin 25 mg daily and subcutaneous liraglutide 0.6 mg in the morning and 1.8 mg at night.

On the third day of admission (day 7 of illness), he became more lethargic and had persistent vomiting. He was diagnosed with DKA as his ABG revealed high anion gap metabolic acidosis, capillary blood glucose 11.9 mmol/L, and urine ketone 3.0 mmol/L. Serum ketone was not available. Other laboratory investigations showed blood urea 11.4 mmol/L, sodium 136 mmol/L, potassium 4.5 mmol/L, chloride 106 mmol/L, and creatinine 105 mmol/L. He was then started on treatment for DKA in the ward with protocolized intravenous fluid administration and fixed-scale insulin infusion, which showed resolution the following day.

On the seventh day of admission (day 11 of illness), the patient developed sudden progressive dyspnea and tachypnea with a respiratory rate of 40 breaths per minute without desaturation, heart rate 110 beats per minute, and stable blood pressure. The ABG taken under room air revealed severe high anion gap acidosis (pH 6.87, pCO_2_ 17 mmHg, pO_2_ 37 mmHg, HCO_3−_ 3.1 mmol/L, lactate 1.7 mmol/L), capillary blood glucose 10.9 mmol/L, and serum ketone 4.2 mmol/L. Treatment for DKA was restarted along with broad-spectrum antibiotics, and he was referred for intensive care unit (ICU) admission in view of possibility of worsening pneumonia. He was started on high-flow mask oxygen therapy while awaiting ICU admission. However, as his chest radiograph did not show worsening COVID-19 pneumonia, steroid therapy was not commenced.

In the ICU, he was put on high-flow nasal cannula (HFNC) oxygen therapy with fraction of inspired oxygen (FiO_2_) 0.5 and flow of 50 L/minute. Protocolized DKA treatment was continued with fixed-scale insulin at 0.1 unit/kg body weight, intravenous fluids, and potassium correction. In addition, sodium bicarbonate infusion was started.

Treatment was adequate during his initial stay in the hospital. There was no issue with compliance with medications. In addition to that, he had been taking orally well prior to his deterioration before ICU admission. It was noted that empagliflozin was continued by the patient himself throughout his stay in the ward, and it was immediately withheld in the Intensive Care Unit (ICU). Severe euglycemic DKA due to empagliflozin was diagnosed in view of high anion gap metabolic acidosis, which could not be explained by the small rise in lactate and ketonemia alone. His urine output remained at more than 0.5 mL/kg/hour throughout his stay, and estimated glomerular filtration rate (eGFR) was persistently more than 50 mL/minute/1.73 m^2^. Serial laboratory investigations taken in ICU are presented in Table 1.

**Table 1:** Initial and follow-up laboratory values

	Prior to ICU admission	Initial (in ICU)	3 hours post-treatment	6 hours post-treatment	9 hours post-treatment	13 hours post-treatment	17 hours post treatment	21 hours post-treatment	24 hours post-treatment	36 hours post-treatment	48 hours post-treatment
Date/time	19/1/21 8.00 am (ABG was taken under room air)	19/1/21 10.39 am (HFNC started)	19/1/21 2.03 pm (CRRT started at 1.45 pm)	19/1/21 17.01 pm	19/1/21 19.53 pm	20/1/21 12.05 am	20/1/21 3.59 am	20/1/21 8.08 am (CRRT clotted at 8.30 am)	20/1/21 10.10 am	20/1/21 22.19 pm	21/1/21 10.08 am
pH	6.87	6.84	6.96	7.19	7.25	7.4	7.43	7.47	7.48	7.49	7.47
HCO_3_ (mmol/L)	3.1	1.5	2.2	3.4	6.1	11.8	18.6	24	23.8	20.6	22.6
pCO_2_ (mmHg)	17	9	10	9	14	19	28	33	32	27	31
pO_2_ (mmHg)	37	250	164	171	167	203	194	183	173	191	111
Lactate (mmol/L)	1.7	1.6	1.0	0.9	0.8	0.9	0.9	1.2	1.0	1.3	0.5
Anion gap (mmol/L)	26	28	27	26	23	17	14	9	9	12	9
Chloride (mmol/L)	–	104	–	–	–	–	107	–	–	–	110
Na/K (mmol/L)	–	133/5.7	–	–	–	–	140/3.0	–	–	–	142/3.5
Hct (%)	–	42	–	–	–	–	33	–	–	–	33
Glucose (mmol/L)	10.9	15	12.9	7.2	6.7	7.9	11.0	16.5	8.5	9.5	12.2
Urea (mmol/L)	–	10.0	–	–	–	–	6.6	–	–	–	6.0
Creatinine (μmol/L)	–	121	–	–	–	–	99	–	–	–	88
Total protein/albumin (g/L)	–	64/40	–	–	–	–	50/30	–	–	–	50/28
Phosphate (mmol/L)	–	1.53	–	–	–	–	0.20	–	–	–	1.04
Serum ketone (mg/dL)	4.2	2.8	–	–	–	–	3.1	–	–	0.6	0.5

*Na* sodium, *K* potassium, *Hct* hematocrit, *ABG* arterial blood gas, *HFNC* high-flow nasal cannula, *CRRT* continuous renal replacement therapy,* pH* potential of Hydrogen,* HCO*_*3*_ serum bicarbonate,* pO*_2_ partial pressure of Oxygen,* pCO*_2_ partial pressure of carbon dioxide

After 3 hours of ICU admission, the patient developed worsening of tachypnea and mild confusion. He required low doses of noradrenaline infusion for about 10 hours to achieve mean arterial pressure (MAP) target of > 65 mmHg, and there was minimal improvement of metabolic acidosis. Subsequently, continuous veno-venous hemodiafiltration (CVVHDF) was commenced to aid in the correction of acidosis. A total of 18 hours of renal replacement therapy was required to achieve normal acid–base balance. The patient continued to improve over the next 24 hours and was weaned down to nasal prong oxygen. Initial partial pressure of oxygen taken under HFNC during his initial stay in ICU was persistently above 150 mmHg (Table 1). We were able to immediately wean off his oxygen requirement within a short period of time, which did not suggest worsening COVID-19 pneumonia. He was transferred out from ICU 3 days later and discharged home after 2 weeks of hospitalization, with modification of his antihyperglycemic agents.

## Discussion

In normal physiology, 90% of the filtered glucose is reabsorbed in the proximal convoluted tubule [4, 6]. SGLT-2 inhibitors are the first class of diabetic medication that act on the proximal convoluted tubule and reduce reabsorption of glucose. The resultant fall in blood glucose levels decreases insulin secretion by the pancreas, leading to an increased glucagon-to-insulin ratio, with an end result of enhanced gluconeogenesis [4]. Insulin deficiency promotes ketogenesis, further leading to ketoacidosis. A minimum of 100 g of carbohydrate is required daily to prevent ketosis [7].

DKA is a serious complication of diabetes that is commonly observed in both T1DM and T2DM patients, especially under extreme physical stress such as trauma, surgery, or infection [2, 8, 9]. Persistent acidosis may be commonly seen after resolution of DKA due to hyperchloremic acidosis from resuscitation with fluids containing zero strong ion difference. In this case, the initial DKA could be triggered by active COVID-19 infection due to high stress level, and continuation of empagliflozin resulted in a rebound of ketosis. Euglycemic DKA can be attributed to multiple confounding factors in this case. However, the most likely cause is empagliflozin usage. This is evidenced by mild COVID-19 pneumonia not requiring steroid therapy, patient compliance to insulin medication during hospitalization, good oral intake, and adequate treatment during first DKA episode. The exact pathophysiology behind the mechanisms involved is yet to be determined. Fasting beta hydroxybutyrate (β-HB) levels are doubled after empagliflozin administration, as reported by Ferranini *et al*. [2, 10], as there is a rise of β-HB due to overproduction and, hence, both clearance rate and fractional excretion of β-HB are increased [2, 11].

Guidelines for treatment of euDKA are not readily available. Relevant recommendations for the treatment of DKA suggest volume restoration with isotonic fluid, ensuring serum potassium > 3.3 mmol/L before initiation of insulin therapy of 0.1 unit/kg body weight, while bicarbonate infusion should be considered in cases of life-threatening acidosis with pH < 6.8 [2, 8].

There is no clear evidence to suggest a role of hemodialysis in removing empagliflozin. The patient information leaflet by Boehringer Ingelheim Pharmaceuticals [12] states that removal of empagliflozin by hemodialysis is yet to be studied. The use of hemodialysis in empagliflozin overdose is unlikely to be of benefit given the drug has a relatively high protein binding of approximately 86.2% [13]. However, continuous renal replacement therapy (CRRT) does play a role in restoring acid–base balance in overwhelming acidemia, as was the case in our patient. Therefore, it may be prudent to start renal replacement therapy early in very severe empagliflozin-related euDKA when the patient does not show signs of improvement or further deteriorates.

## Conclusions

Diagnosis of euDKA secondary to empagliflozin in a patient with COVID-19 can be easily missed. Health care workers should have a high index of suspicion while managing a patient with COVID-19 presenting with atypical DKA taking SGLT-2 inhibitors, which has become a popular medication among T2DM. Early detection, withholding the inciting agent, and instituting appropriate and timely treatment are necessary to avoid further untoward consequences during the COVID-19 pandemic.

## Data Availability

This is a case report of a clinical case; all information relevant to the clinical course of the patient remains encrypted at our hospital’s archive and is not available outside our hospital.

## Abbreviations

EuDKA: Euglycemic diabetic ketoacidosis
T1DM: Type 1 diabetes mellitus
DKA: Diabetic ketoacidosis
HCO_3−_: Serum bicarbonate
SGLT-2: Sodium–glucose cotransporter
FDA: Food and Drug Administration
T2DM: Type 2 diabetes mellitus
EMA: European Medicines Agency
ABG: Arterial blood gas
HFNC: High-flow nasal cannula
FiO_2_: Fraction of inspired oxygen
eGFR: Estimated glomerular filtration rate
CVVHDF: Continuous venovenous hemodiafiltration
β-HB: Beta hydroxybutyrate
CRRT: Continuous renal replacement therapy
